# RNA activation as a precision dosing modality: MTL-CEBPA for controlled enzyme elevation in MPS I-H

**DOI:** 10.3389/fmed.2026.1813362

**Published:** 2026-05-07

**Authors:** Vikash Reebye, Konstantina Skourti-Stathaki, Konstantinos Vanezis, Minsun Song, Michael J. Przybilla, Karin Purdie, Amelia J. Tesone, Grazia Pizza, Sheba Jarvis, Nina Raulf, Pinelopi Andrikakou, Jenni Vasara, Ryan Setten, Joanna Nicholls, Siv Anita Hegre, Päl Sætrom, Robert Habib, Kai-Wen Huang, Farzin Farzaneh, Haitang Li, John J. Rossi, Shunji Tomatsu, Shaukat Khan, Laura Furness, Robert Wynn, Chester B. Whitley, Simon A. Jones, Nagy A. Habib

**Affiliations:** 1 Department of Surgery and Cancer, Imperial College London, London, United Kingdom; 2 MiNA Therapeutics Limited, London, United Kingdom; 3 Dawn Therapeutics Limited, London, United Kingdom; 4 Department of Molecular & Cellular Biology, Beckman Research Institute of City of Hope, Duarte, CA, United States; 5 Division of Pediatric Blood and Bone Marrow Transplantation, Minneapolis, MN, United States; 6 Department of Cancer Research and Molecular Medicine, Norwegian University of Science and Technology, Trondheim, Norway; 7 Department of Computer and Information Science, Norwegian University of Science and Technology, Trondheim, Norway; 8 National Taiwan University Hospital, Taipei, Taiwan; 9 The Rayne Institute, King’s College London, London, United Kingdom; 10 Skeletal Dysplasia Research Laboratory, Nemours/Alfred I DuPont Hospital for Children, Wilmington, DE, United States; 11 Manchester Centre for Genomic Medicine, St Mary’s Hospital, Manchester University NHS Foundation Trust, Manchester, United Kingdom; 12 Department of Pediatric Bone Marrow Transplantation, Manchester University NHS Foundation Trust, Manchester, United Kingdom

**Keywords:** α-L-iduronidase, enzyme deficiency, Hurler-Scheie syndrome, MPS-IH, RNA activation, small activating RNA, therapeutic oligonucleotides

## Abstract

**Background:**

Gene therapy and hematopoietic stem cell transplantation (HSCT) have transformed outcomes for severe mucopolysaccharidosis type I (MPS I-H), yet a critical unmet need remains. Children with MPS I-H frequently experience progressive skeletal, cardiac, and other complications despite timely HSCT, largely because enzyme activity cannot be safely and precisely titrated over time. Irreversible genetic modification via integrating vectors offers supra-physiological enzyme levels but carries long-term safety and re-dosing liabilities in patients treated early in life.

**Methods:**

We investigated RNA activation (RNAa) as a precision dosing strategy to enhance endogenous IDUA expression without permanent genome alteration. Using MTL-CEBPA, a small activating RNA that upregulates CEBPA transcription factor, we characterized CEBPA–IDUA relationships *in vitro*, *in vivo*, and in legacy clinical samples from cancer patients.

**Results:**

CCAAT enhancer binding protein alpha activation consistently increased IDUA mRNA across A549, IMR90, and mesenchymal stem cells. In wild-type mice, two intravenous MTL-CEBPA doses produced a ∼2-fold, durable increase in bone marrow IDUA mRNA and plasma enzyme activity, sustained for up to 4 weeks. In humanized bone marrow–transplanted MPS I-H mice, repeated dosing with MTL-CEBPA led to an approximately 2-fold increase in circulating IDUA activity compared with controls over the 3-weeks treatment period. The largest apparent separation from controls was observed in the homozygous cohort, although these genotype-specific differences should be interpreted cautiously given the limited subgroup sizes. In cancer patient–derived monocytes, increased CEBPA protein levels correlated with higher IDUA levels (R^2^ = 0.571). Consistent with this, approximately half of evaluable patients exhibited increased plasma IDUA activity following treatment.

**Conclusion:**

These translational data demonstrate that MTL-CEBPA delivers controlled, reversible enhancement of IDUA in the context of HSCT, providing robust pharmacodynamic proof-of-concept rather than definitive evidence of durable efficacy. By enabling titratable enzyme elevation without integrating vectors, RNAa therapeutics address a key unmet need in Hurler syndrome: safe fine-tuning of residual enzyme activity over a patient’s lifetime. With scalable, cost-effective oligonucleotide manufacturing, MTL-CEBPA and related RNAa therapeutics represent a clinically relevant adjuvant strategy for HSCT-treated MPS I-H, with potential for other enzyme deficiency disorders.

## Introduction

Mucopolysaccharidosis type I (MPS I) is a rare, autosomal recessive lysosomal storage disorder caused by deficiency of the enzyme α-L-iduronidase (IDUA). This leads to accumulation of the glycosaminoglycans (GAGs), heparan sulfate and dermatan sulfate in lysosomes, resulting in multisystem organ dysfunction. Clinical features include cardiac valve thickening, cardiomyopathy, respiratory disease, cognitive decline, hepatosplenomegaly, skeletal dysplasia, and vision impairment. MPS I is classified by severity into severe, MSP I-H (Hurler syndrome), intermediate MPS I-H/S (Hurler-Scheie), and attenuated MPS I-S (Scheie) forms, based on residual enzyme activity, age of onset, and clinical phenotype. Untreated, severe MPS I-H (Hurler syndrome) leads to death within the first decade, often earlier. Attenuated forms present later and progress more slowly, with variable life expectancy (1).

Currently, two main treatments are available for MPS I-H: enzyme replacement therapy (ERT) and hematopoietic stem cell transplantation (HSCT) (2).

Enzyme replacement therapy involves weekly infusions of recombinant α-L-iduronidase, which effectively improve somatic symptoms affecting the heart, lungs, liver, spleen, and kidneys (3, 4). However, ERT cannot cross the blood–brain barrier and shows limited efficacy in poorly vascularized tissues such as cartilage and heart valves. Consequently, it fails to prevent neurological decline or skeletal disease progression. Long-term use can also elicit anti-IDUA antibodies, reducing therapeutic benefit and occasionally causing immune reactions (5–7).

Hematopoietic stem cell transplantation is recommended for patients diagnosed before 2.5 years of age, ideally before irreversible pathology develops, and is often used in combination with ERT (2). Donor-derived cells provide a continuous source of enzyme, promoting cross-correction of deficient cells through the mannose-6-phosphate receptor pathway. Although HSCT can partially address central nervous system involvement by supplying enzyme-secreting cells to the brain (8), it often fails to fully correct lysosomal storage in avascular tissues such as bone, cartilage, and cornea. Consequently, skeletal, cardiac, and visual complications may persist despite successful engraftment, representing a major residual disease burden (9–12).

Post-transplant outcomes correlate closely with leukocyte IDUA activity: higher enzyme levels are associated with reduced substrate deposition and fewer disease-related surgical interventions (10). Yet, even with wild-type donor grafts, long-term follow-up studies show that substantial morbidity–including skeletal dysplasia and sensory deficits–progresses over time (9, 12). Lentiviral hematopoietic stem-cell gene therapy has emerged as an important extension of this approach, capable of achieving supra-physiological enzyme expression (13).

Nonetheless, a clinical need remains for complementary, adjustable strategies to enhance IDUA activity post-transplant, particularly where enzyme demand changes with age, growth, or tissue-specific pathology. These considerations motivated us to investigate a systemically administered, titratable approach to augment IDUA expression after transplantation.

To identify upstream regulators of IDUA expression, we performed in silico PROMO analysis of its 5’ regulatory region which revealed putative CEBPA binding motifs, indicating that CEBPA may directly influence IDUA transcription (Supplementary Figure 1). This finding provided the mechanistic rationale for repurposing MTL-CEBPA, a clinically validated small activating RNA (saRNA) originally developed as an immunomodulatory agent in hepatocellular carcinoma, to enhance IDUA expression in MPS I-H. Here, we present preclinical and translational evidence that MTL-CEBPA increases bone marrow IDUA transcription and plasma enzyme activity in MPS I-H models and human samples, highlighting RNA activation (RNAa) as a precision, non-integrating modality that can be layered onto HSCT to address residual disease in Hurler syndrome.

## Materials and methods

We designed a series of *in vitro*, *in vivo*, and translational analyses to test whether pharmacological upregulation of CEBPA using the saRNA drug MTL-CEBPA can achieve dose-dependent, reversible increases in *IDUA* expression and enzyme activity across relevant preclinical systems and in human clinical samples.

### Cell culture and transfection

A549 cells, IMR90 cells, and MSCs (ATCC) were grown in ATCC-formulated RPMI medium supplemented with 10% fetal bovine serum following manufacturer’s protocols. Cells were maintained in a 5% CO_2_ incubator. Unless otherwise specified, for transfections, the cells were seeded at 1.5 × 10^5^ cells per well in a 24-well plate and reverse transfected immediately after seeding with the indicated oligonucleotide concentration using 1 uL per well of Lipofectamine 2000 (Life Technologies).

### *In silico* PROMO analysis

PROMO was used to screen the 5′ regulatory DNA sequence of IDUA gene.^1^

### RNA isolation and qPCR from cell lines

RNA was isolated from cultured cells using the RNeasy Mini Kit (QIAGEN). RNA was quantitated using a QiaXPERT spectrophotometer (Qiagen), and 500 ng was reverse transcribed using the Quantitect Reverse Transcription Kit (QIAGEN). Relative expression levels were determined by qPCR using Quantifast SYBR Green Master Mix (QIAGEN) on an QuantStudio thermal cycler (LifeTechnologies). Quantitect Primer Assays (QIAGEN) were used to probe for transcript levels of IDUA and Actin B as the housekeeping gene. Relative expression was determined using the standard Livak method (2^–ΔΔCt/*sup method*^) normalized to GAPDH expression.

### Assessment of MTL-CEBPA in wild-type mice

All animal work was conducted in accordance with UK Home Office legislation and approved institutional guidelines. Male C57BL/6 mice (6 weeks old) were obtained from Charles River UK and assigned a unique tag identifier upon arrival. Sample size calculations were performed *a priori* based on variance estimates from prior studies to ensure adequate statistical power for detecting expected treatment effects. Animal allocation to treatment groups was randomized using a computer-generated random number sequence based on tag identifiers, and investigators were blinded to treatment assignment during dosing and analysis. Mice received two intravenous tail-vein injections, 24 h apart, of either MTL-CEBPA or NOV340-FLUC at 2 mg kg^1^ [formulated CEBPA-51 (saCEBPA) and siFLUC as previously described (14–17)]. The animals were weighed three times weekly. Bone marrow samples were taken at termination. The left femur was excised and the femoral cells flushed out using 1 mL PBS. An equal volume of 70% ethanol was added and the solution was mixed by pipetting. The mixture was then transferred to RNeasy columns following the manufacturer’s instructions. After the wash steps, 30 ul of water was used for eluting the RNA from the column. RNA Samples were quantified using the QIAGEN QIAxpert spectrophotometer according to the manufacturer’s protocol. QC of the samples was performed using the ScreenTape Assay for Tapestation (Agilent) according to the manufacturer’s recommended protocol. Samples with RIN greater than 7.0 were considered as meeting the threshold quality for qPCR amplification. Samples were subjected to reverse transcription using the Qiagen Quantitect RT Kit with 300–500 ng RNA input which was standardized across the samples. Reverse transcription was performed according to the manufacturer’s protocol. Oligo-dT primer (QIAGEN) was substituted for the default random hexamer/oligo-dT primer mix in the kit. In brief, for the RT reaction, 2 μL of the gDNA wipeout was added to 12 ul diluted RNA for 2 min at 42°C followed by 1 μL RT enzyme, 1 μL oligodT primer, and 4 μL 5xRT buffer for a total of 20 μL each sample. Following the RT step, 140 μL of water was added to each cDNA sample for an 8-fold dilution prior to qPCR amplification.

### qPCR

SYBR qPCR was performed for the analysis of CEBPA gene expression. Relative gene expression was measured in cDNA samples by qPCR using the ThermoFisher PowerUp SYBR Green Master Mix on a QuantStudio 5 qPCR machine using QIAGEN QuantiTect murine primer assays for CEBPA and 4 housekeeping genes: *GAPDH*, *HPRT*, *B2M*, *ACTB*. Reactions were run in triplicate with each 10↾L reaction volume composed of the following: 1↾L primer assay, 5↾L 2× SYBR Green mastermix, 4↾L diluted cDNA from above. The following cycling conditions were used: initial heat activation step 2 min @95 *^o^*C then 40 cycles comprising 1 s @95 *^o^*C, 30 s @60 *^o^*C followed by melt curve analysis. geNORM analysis revealed that the most consistent housekeeping genes for these samples were HPRT and B2M so a mean of these 2 was used for normalization. Taqman qPCR was performed for the analysis of *IDUA* mRNA expression. Relative mRNA expression of *IDUA* was measured in cDNA samples by qPCR using the Thermo Fisher TaqMan Fast Advanced Master Mix according to the manufacturer’s protocol on a QuantStudio 5 qPCR machine with a Life Tech Taqman assay for murine *IDUA*. Reactions were run in triplicate with each 10↾L reaction volume composed of the following: 0.5↾L primer assay, 5↾L 2× SYBR Green mastermix, 4↾L diluted cDNA from above, 0.5↾L water. The following cycling conditions were used: initial heat activation step 20 s @95 *^o^*C then 40 cycles comprising 1 s @95 *^o^*C, 20 s @60 *^o^*C. The maximum amount of blood (600↾L–800↾L) was collected by cardiac puncture and transferred to K2 EDTA treated tubes. Tubes were spun down at 300 g for 5′ at 4^°^C and the plasma aliquoted and stored at −80 ^°^C until analysis.

### IDUA enzyme activity assay

#### Preparation of serum from blood samples

After blood was collected and allowed to clot, serum was separated following standard centrifugation guidelines (5000 rpm for 15′). In duplicate, 25 μL of serum was mixed with 25 μL of 360 μM 4-methylumbelliferyl alpha-L-iduronide (Glycosynthe) in 0.4M Formate buffer at pH3.5 in a microplate. Sample blanks were prepared by mixing 25 μL 0.2% BSA in PBS with 25 μL of 360 μM 4-methylumbelliferyl alpha-L-iduronide in 0.4M Formate buffer at pH 3.5. A 10-point standard curve of 4-methylumbelliferone in 0.4M Formate buffer at pH 3.5 ranging from 0.04 to 0.4 ug/mL was prepared at the same time. Plates were then protected from light and incubated for 30′ at 37 ^°^C. The reaction was stopped by the addition of 200 μL of glycine carbonate buffer. Samples were then read in a Biotek plate reader at 365 nm excitation and 450 nm emission. Enzyme activity was calculated by dividing activity in ng/h by mL of serum using the formula: Activity in nmol/h/ml where specific activity was summarized as ng/h/ml divided by FW of 4MU (176.17).

### *In vivo* assessment of MTL-CEBPA in humanized MPS-I-H mice

#### Generation of the MPS I-H disease mouse model

To generate the MPS I-H mouse model, breeding challenges were encountered due to reproductive limitations. Females homozygous for all three mutations (Idua^*W*392*X*^, scid, and Il2Rγ*^null^*) exhibited poor maternal behavior, while males homozygous for the Idua^*W*392*X*^ mutation, homozygous for scid, and hemizygous for the X-linked Il2Rγ*^null^* mutation demonstrated subfertility, leading to a higher incidence of non-productive breeding pairs. To maintain a viable colony, breeding strategies involved crossing females heterozygous for the Idua^*W*392*X*^ mutation and homozygous for the scid and Il2Rγ*^null^* mutations with males heterozygous for the Idua^*W*392*X*^ allele, homozygous for the scid mutation, and hemizygous for the X-linked Il2Rγ*^null^* mutation. NOD.Cg-Idua^TM1*Clk*^ Prkdc*^scid^* Il2rg^TM1*Wjl*^/J (NSG-MPS I-H) heterozygous mice were obtained from Jackson Laboratories and incorporated into the breeding strategy.

#### Genotyping by rhAmp SNP assays

Total genomic DNA was extracted from mouse tail samples (5 mm of tail placed into a 1.5 mL tube) using DirectPCR Lysis Reagent (VivaGen Biotech), following the manufacturer’s protocol. For extraction, 200 μL of DNA lysis buffer and 2 μL of 20 mg/mL Proteinase K solution were added to each sample. The samples were incubated overnight at 55 °C and then heated at 85 °C for 45 min to inactivate Proteinase K. DNA was purified via phenol/chloroform extraction and ethanol precipitation, and the concentration was determined. Purified DNA was stored at −20 °C until use. Genotyping was performed using rhAmp technology, which employs dual-enzyme chemistry based on RNase H2-dependent PCR (rhPCR) and universal reporters. Each assay utilized two allele-specific primers along with a locus-specific primer. Synthetic gBlocks™ gene fragments representing known genotypes served as controls for each SNP assay: one for the wild-type allele (WT), one for the mutant allele (MA), and a 1:1 mixture to represent the heterozygous genotype. Primers were designed using the rhAmp^®^ Genotyping Design Tool (IDT)^2^ as detailed in Table 1. The rhAmp genotyping assay was performed according to the manufacturer’s instructions. Briefly, 2 μL (10 ng) of DNA sample was mixed with 5.3 μL of rhAmp Genotyping Mix [1 mL of rhAmp Genotyping Master Mix 2× (Cat. no. 1076017; IDT) and 50 μL of rhAmp Reporter Mix (Cat. no. 1076028; IDT)] and 0.5 μL of custom rhAmp SNP assays (IDT), achieving a final volume of 10 μL. Standard samples and negative template controls used in the HRM analysis were also included.

**TABLE 1:** Primers for mouse genotyping.

Primer type	Sequence 5′ 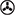 3′	Quencher
Common	CCT GGG GAT TCC TTC CAC	
Wild type reverse	GGC ACA GTG ACC CAG GAG	
Mutant reverse	GGC TCT ATG GCT TCT GAG G	
WT probe	CTG CCA AGG TCA CCA ATG T	FAM
MUT probe	CCT GCC AAG GTC ACG AG	HEX

The SNP genotyping assays were prepared with 0.25 μL of rhAmp SNP Assays (20×), 2.65 μL of combined rhAmp Genotyping Master Mix (2×) and rhAmp Reporter Mix (40×), 0.10 μL of nuclease-free water, 2 μL of sample DNA, and 2 μL of control template (gBlocks fragment controls) or nuclease-free water for no-template controls. Reactions were conducted on a CFX Connect Real-Time PCR Detection System (Bio-Rad Laboratories, Hercules, CA, USA), and data were analyzed using CFX Maestro Software version 2.3 (Bio-Rad). Thermal cycling conditions followed the standard protocol: 95 °C for 10 min, followed by 40 cycles at 95 °C for 10 s, 60 °C for 30 s, and 68 °C for 20 s.^3^

Bi-allelic specificity was achieved using two probes, one labeled with Amidite-fluorescein (FAM™) dye and the other with Hexachloro-fluorescein (HEX™) dye (Table 2). These dyes were detected independently with appropriate excitation sources and emission filters at their respective wavelengths. All genomic DNA samples were quantified, and each specimen was classified as resistant (RR), susceptible (SS), or heterozygous (RS) based on the melting temperature obtained. The results were displayed as puncta on an XY scatter plot based on relative fluorescent units (RFU), facilitating allelic discrimination through CFX Maestro software (Bio-Rad Laboratories).

**TABLE 2:** Different excitation and emission spectra for fluorophores.

Quencher	Excitation nm	Emission nm
FAM	450	533
HEX	483	568

### Ethics statement

All animal care and experimental procedures were conducted in accordance with protocols reviewed and approved by the City of Hope Institutional Animal Care and Use Committee (IACUC), under the principal investigator for this study, John Rossi (IACUC 23017). Human fetal liver tissue was obtained from Cercle Allocation Services, Inc., a non-profit organization, in compliance with all applicable federal and state regulations. The vendor maintains its own Institutional Review Board (IRB) and adheres to human subject protection requirements.

### Humanized mouse generation

To generate humanized NSG-MPS I-H mice, two preparative methods were employed. First, 5–8-weeks-old NSG-MPS I-H mice were irradiated with 230 cGy and subsequently transplanted via intravenous (i.v.) injection with 2 × 10^5^–1 × 10^6^ CD34+ hematopoietic stem cells (HSCs) isolated from human fetal liver tissue. Alternatively, a second approach involved administering intraperitoneal (IP) injections of busulfan (35 mg/kg, adjusted by weight) for four consecutive days. On the fifth day, 130,000 CD34+ human fetal liver stem cells were injected through the tail vein to establish humanization. Tail vein injections were performed using sterile strainers, with proper disposal of needles in biohazard containers without recapping. Engraftment was monitored through retro-orbital blood collection, and mice were observed for 10–20 weeks post-transplantation. At 12 weeks, engraftment was evaluated by flow cytometry analysis of peripheral blood samples.

### Blood collection and analysis

Baseline blood samples were collected for IDUA analysis. Subsequent blood samples were collected weekly for a duration of 7 weeks. The blood plasma IDUA enzyme activity was assessed using the 4-methylumbelliferyl-α-L-iduronide (4Mu) enzyme colorimetric assay.

### Statistical methods

GraphPad Prism (version 8.1.1, GraphPad Software, Inc.) was used to graph and analyze all data. *In vitro* transfection data was analyzed for saCEBPA vs. Fluc comparison using unpaired *t*-test with Welch’s correction. *In vivo* mouse and white blood cell patient data were analyzed by two-way ANOVA.

## Results

### CEBPA activation enhances IDUA expression across multiple cell types

To further characterize the regulatory relationship between CEBPA and IDUA suggested by the *in silico* PROMO analysis, we investigated IDUA transcriptional control across distinct cellular contexts using both transformed (A549) and non-transformed (IMR90) human lung cell lines. We employed the small activating RNA (saRNA) CEBPA51 (saCEBPA), previously developed to upregulate CEBPA expression (14), and transfected A549 cells to assess whether CEBPA functions as a direct activator of IDUA transcription.

**FIGURE 1:**
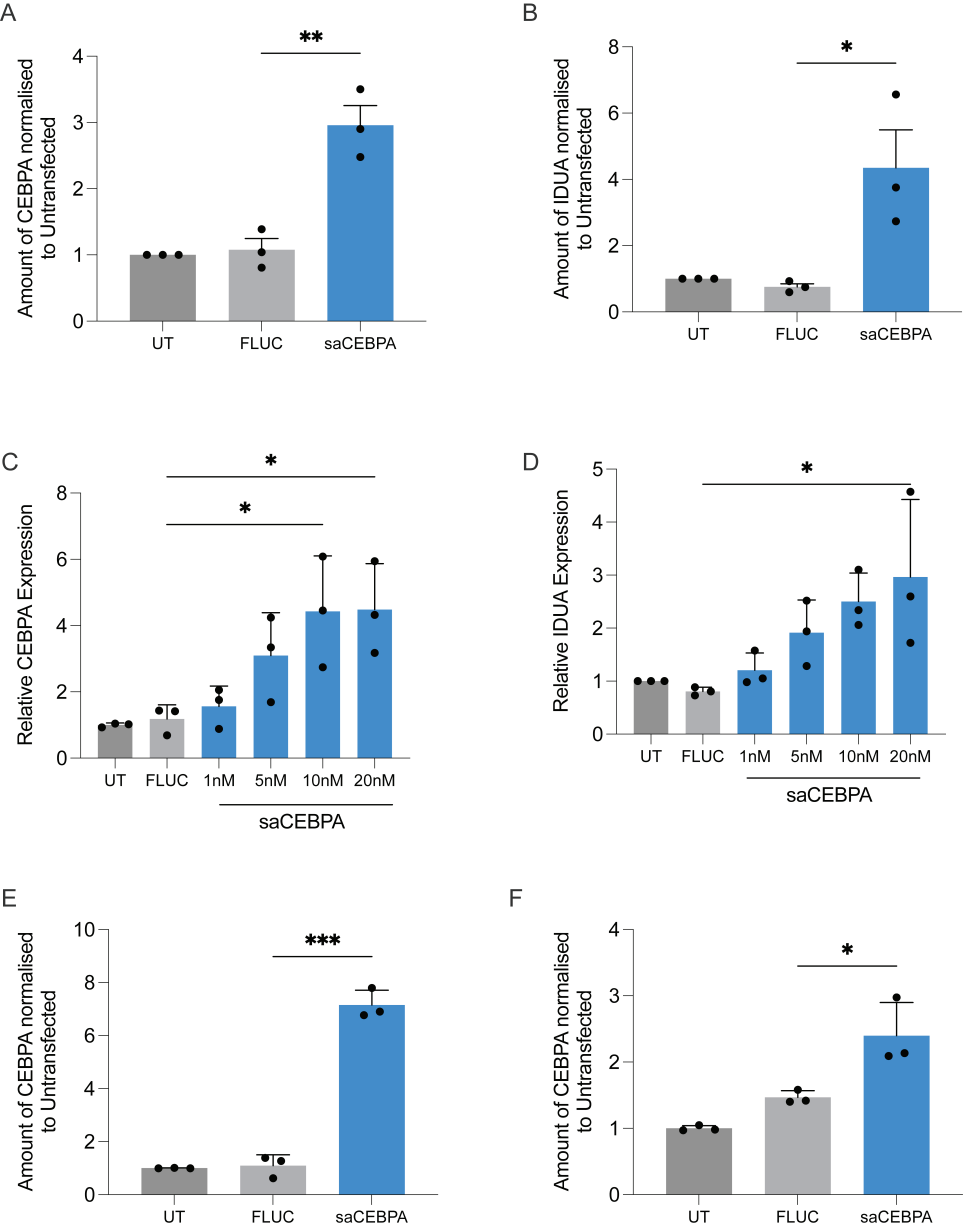
saCEBPA transcriptionally activates IDUA. **(A)** qRT-PCR for RNA from A549 cells showing CEBPA mRNA increases following treatment with saCEBPA relative to control (FLUC) and untreated cells. **(B)** qRT-PCR for RNA from A549 cells showing IDUA mRNA increases following treatment with saCEBPA relative to control (FLUC) and untreated cells. **(C)**. qRT-PCR for RNA from IMR90 fibroblast cells showing CEBPA mRNA increases following treatment with saCEBPA relative to control (FLUC) and untreated cells at a range of doses. **(D)** qRT-PCR for RNA from IMR90 cells showing IDUA mRNA increases following treatment with saCEBPA relative to control (FLUC) and untreated cells. **(E)** mRNA analysis of CEBPA in MSC cells at 196 h relative to control (FLUC) and untreated cells. **(F)** mRNA analysis of IDUA in MSC cells at 196 h relative to control (FLUC) and untreated cells in the same conditions as above. *N* = 3 biological replicates. Error bars are ±SD and statistics are performed using unpaired *t*-tests (*p < 0.05, **p < 0.01, and ***p < 0.001). In all figure panels saCEBPA denotes CEBPA-51.

This resulted in a 3-fold increase in *CEBPA* mRNA levels (Figure 1A) and a corresponding 4-fold increase in *IDUA* mRNA expression (Figure 1B) relative to control (FLUC). We then investigated the transcriptional effect of saCEBPA on *IDUA* mRNA expression in non-transformed IMR90 cells. Cells were transfected with increasing doses of saCEBPA (1nM, 5nM, 10nM, and 20nM). FLUC control was transfected at 20nM. mRNA expression was assessed by RT-PCR at 72 h post-transfection for both *CEBPA* and *IDUA*. Results showed *CEBPA* mRNA expression was significantly upregulated at 10nM and 20nM saCEBPA. Values were normalized to untreated cells (Figure 1C). *IDUA* mRNA expression also increased within the same dose range of 10nM to 20nM of saCEBPA (Figure 1D). To further explore the transcriptional relationship, we assessed the effect in mesenchymal stem cells. We observed increased mRNA expression of both *IDUA* and *CEBPA*, which persisted for 7 days (Figures 1E, F). These findings demonstrated that *CEBPA* activation consistently leads to increased *IDUA* mRNA expression across multiple cell types, including transformed lung cells, non-transformed lung fibroblasts, and mesenchymal stem cells, with a clear dose–response relationship and sustained expression over several days after a single exposure. This behavior is consistent with a pharmacologically titratable transcriptional control mechanism rather than permanent genetic modification.

#### *In vivo* evidence that MTL-CEBPA increases IDUA expression and activity

**FIGURE 2:**
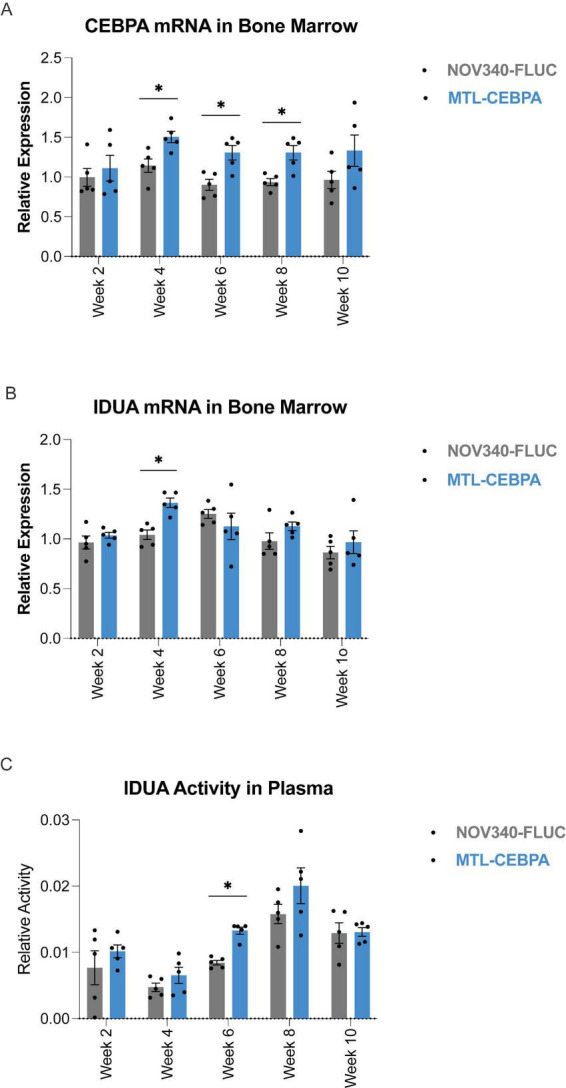
Durable increase of IDUA expression and activity in wild-type mice treated with MTL-CEBPA. **(A)** CEBPA mRNA expression in bone marrow of mice treated with MTL-CEBPA at baseline and then followed bi-weekly for 10 weeks. **(B)** IDUA mRNA expression in the bone marrow and **(C)** IDUA enzyme activity in the plasma of MTL-CEBPA treated mice for a period of 10 weeks. Animals were treated with 2 × 2 mg/kg infusions at baseline, 24 h apart. *N* = 5 mice per group. Error bars are -/ + SD and statistics are performed using unpaired *t*-tests (*p < 0.05).

To evaluate the *in vivo* pharmacodynamic effect of saCEBPA on upregulation of IDUA enzyme activity, wild-type C57/BL6 female mice were administered 2× bolus injection of 2 mg/kg of MTL-CEBPA via tail vein IV infusion over a 24-h period so that the animals would have received a total of 4 mg/kg of the RNAa (*n* = 5 for each group). Effects of MTL-CEBPA were measured over a period of 10 weeks. The pharmacodynamic activity of MTL-CEBPA was determined by measuring *CEBPA* and *IDUA* mRNA in the bone marrow and IDUA activity in plasma with a fluorometric assay using 4-methylumbelliferyl alpha-L-iduronide (4-MU iduronide) as substrate. As shown in Figure 2A, there was a significant increase in *CEBPA* mRNA expression at 4-, 6-, and 8-weeks post-administration relative to control. A significant increase in *IDUA* mRNA in bone marrow was observed at week 4 (Figure 2B). Interestingly, we observed that the increase in *IDUA* mRNA expression was correlated with an increase in IDUA activity in plasma at 6 weeks post treatment (Figure 2C). Collectively, these data provide *in vivo* proof-of-concept that systemic IV administration of MTL-CEBPA can produce a sustained, approximately two-fold elevation in *IDUA* expression and circulating enzyme activity after a short course of dosing. Importantly, the effect gradually wanes over several weeks, indicating that enzyme activity can, in principle, be modulated over time through dosing frequency and amount, without permanent genomic alteration.

#### *In vivo* effects of MTL-CEBPA in humanized bone marrow transplanted MPS I-H mice

To explore the potential for MTL-CEBPA to be used as a treatment to enhance the efficacy of bone marrow transplant in MPS I-H mice, we assessed the benefits of MTL-CEBPA treatment in NSG-MPS I-H mice (Jackson Lab). 8-weeks old NSG-MPS I-H mice were irradiated with 230cGy and subsequently underwent i.v transplantation with healthy CD34^+^ hematopoietic stem cells (HSCs) isolated from human fetal liver tissue. Following successful engraftment confirmation (>10% hCD45+ cells in blood at 18 weeks post-transplantation, Supplementary Figure 2), we initiated a twice-weekly MTL-CEBPA treatment regimen to assess its long-term effects over a 3-weeks period.

**FIGURE 3:**
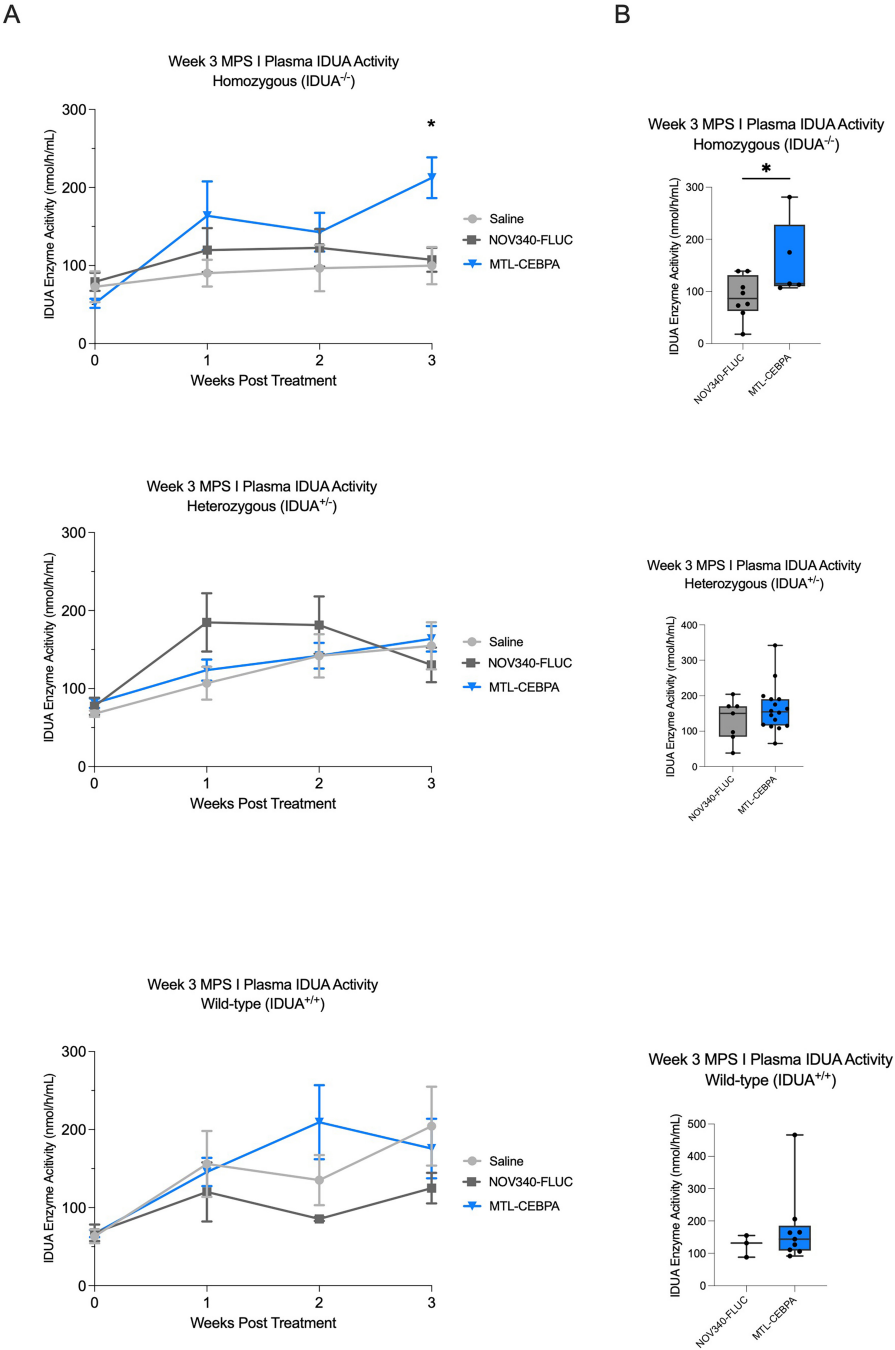
Durable increase of IDUA enzymatic activity in MPS I-H following human CD45 engraftment. IDUA enzyme activity in the serum of humanized wild-type, heterozygous, and homozygous mice MPS I-H mice were measured following baseline infusion of 4 mg/kg MTL-CEBPA **(A)**. Summary at Week 3 of IDUA enzyme activity **(B)**. Data are relative to saline treated and MTL-FLUC treated animals. Data are expressed as mean ± SD of the animals in each group. Error bars are -/ + SD and statistics are performed using unpaired *t*-tests (*p < 0.05).

Compared to the control groups (saline and NOV340-FLUC), animals in the MTL-CEBPA treated groups exhibited elevated IDUA activity levels. While the increase was modest in weeks 1 and 2, IDUA activity peaked at week 3, reaching levels at least twofold higher than the control groups (158 nmol/h/mL in the MTL-CEBPA arm versus 88 nmol/h/mL in the NOV340-FLUC arm), Figure 3A. The apparent magnitude of the MTL-CEBPA-associated increase in circulating IDUA activity differed among genotype groups by the end of the 3-weeks study period, with the largest separation from control observed in the homozygous cohort. In contrast, the heterozygous and wild-type groups showed a smaller separation between treatment and control, likely reflecting higher background enzyme activity and a narrower dynamic range in these cohorts (Figure 3B).

Differential responses across wild-type, heterozygous, and homozygous cohorts indicate a larger pharmacodynamic effect in animals with lower baseline enzyme levels, with caution warranted due to limited subgroup sizes. In the clinically relevant setting of HSCT-treated Hurler patients, these data support the more limited conclusion that RNAa can increase circulating IDUA activity in a post-transplant context and may be particularly informative in settings of low baseline enzyme reserve, highlighting the need for larger, longer-term studies.

### Relationship between *CEBPA* and *IDUA* in clinical trial samples

To show clinical proof of concept that MTL-CEBPA administered IV to a human can lead to increased *IDUA* mRNA expression and IDUA enzyme activity in the circulation, human monocyte samples from legacy clinical studies [NCT02716012] that investigated the effects of MTLCEBPA in cancer patients, were leveraged and examined to determine the relationship between CEBPA and IDUA (17, 18).

**FIGURE 4:**
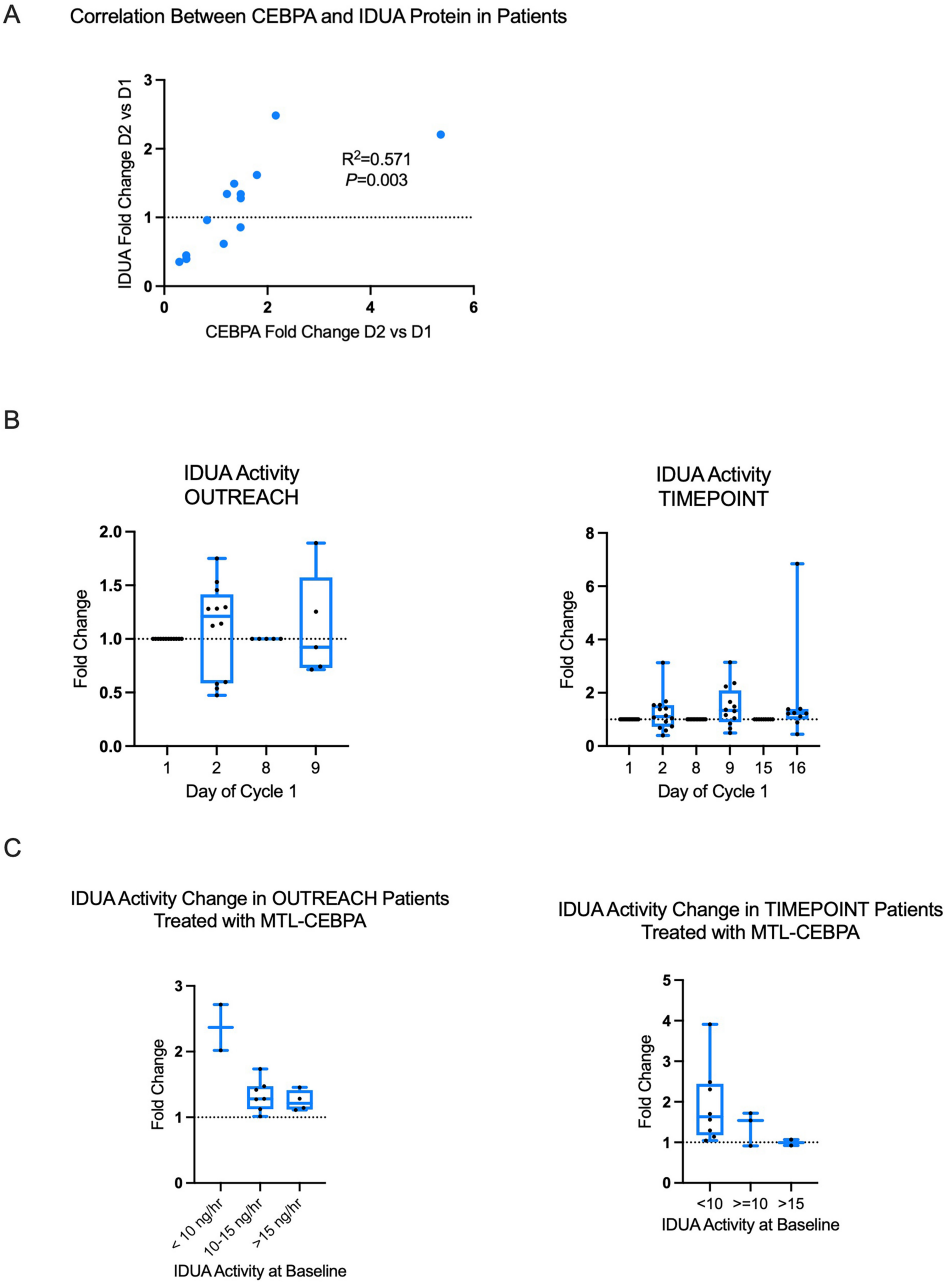
Increases in IDUA enzymatic activity in cancer patients following treatment with MTL-CEBPA. Correlation between IDUA protein and CEBPA protein measured in WBCs from patients 24 h post treatment with MTL-CBEPA **(A)**. Comparison of IDUA activity in patients enrolled in the TIMEPOINT and OUTREACH clinical trials. Treatment infusions are on Day 1 and Day 8 **(B)**. Representation of changes in IDUA activity in patients categories based on baseline IDUA activity **(C)**.

Levels of IDUA and CEBPA protein were measured in monocytes samples collected from the TIMEPOINT clinical trial (17), where a positive correlation was observed (R^2^ = 0.571) (Figure 4A). Further, legacy plasma samples from patients enrolled in the OUTREACH and TIMEPOINT clinical trial were tested for IDUA activity pre- and post-MTL-CEBPA administration (Figure 4B; note: due to biospecimen availability it was not possible to measure every timepoint in each patient). Approximately half of the patients had an increase in IDUA activity post MTL-CEBPA. Finally, we observed a trend that potentially indicates that patients with the lowest baseline plasma IDUA activity had the greatest increase (Figure 4C). Together, these analyses demonstrate that MTL-CEBPA can modulate IDUA biology in humans using standard intravenous dosing schedules already shown to be safe and tolerable in oncology, and that patients with lower baseline IDUA levels may derive the largest pharmacodynamic gain. This provides a clinically relevant starting point for dose-finding strategies aimed at titrating enzyme elevation in HSCT-treated MPS I-H patients.

## Discussion

Despite major advances in HSCT and enzyme replacement therapy, Hurler syndrome remains an archetypal example of an enzyme deficiency disease in which current modalities cannot deliver optimally tuned, life-long enzyme activity. Many children who undergo HSCT early in life still develop progressive skeletal disease, valvular pathology, airway compromise, and other complications that materially affect function and quality of life. Studies show that even modest differences in leukocyte IDUA activity translate into clinically meaningful differences in GAG accumulation and surgical burden. In this setting, even relatively modest increases in systemic IDUA have been associated with measurable differences in GAG storage and clinical trajectory in patients receiving HSCT and/or enzyme replacement. Thus, interventions that can reproducibly raise enzyme activity above a low post-transplant baseline by as little as two-fold are biologically plausible candidates to meaningfully influence long-term outcomes, particularly in individuals who remain close to the threshold at which residual IDUA becomes limiting.

A central therapeutic gap therefore persists in Hurler syndrome: despite major advances in HSCT and the emergence of lentiviral gene therapy, many patients continue to experience clinically meaningful residual disease over time. This distinction is supported by long-term follow-up studies in MPS I-H. Aldenhoven et al. reported in an international multicenter study of 217 successfully engrafted patients that a “considerable residual disease burden” remained in the majority of transplanted individuals (9). Similarly, Gardin et al. reported that, after a median follow-up of 9 years, skeletal dysplasia as well as vision and hearing impairment progressed despite HSCT, with significant disability (12). In this context, our intention is not to diminish the importance of LV-based gene therapy, which represents a major advance in the field, but to address a different translational question: whether IDUA activity can be further and flexibly augmented after transplantation using a non-integrating pharmacologic approach.

Our research identified CEBPA as a novel transcriptional regulator of IDUA. RNA activation experiments confirmed this relationship across cellular models, showing *CEBPA* mRNA upregulation enhances IDUA activity in human fibroblasts and mesenchymal stem cells.

Building on prior development of MTL-CEBPA (CEBPA-51 RNAa encapsulated in NOV340 liposomes) for HCC, we tested its potential in MPS I-H models. In humanized bone marrow-transplanted MPS I-H mice, MTL-CEBPA increased circulating IDUA activity over the 3-weeks treatment period compared with control-treated animals. Importantly, the larger apparent effect observed in the homozygous cohort may reflect both greater dependence on donor-derived enzyme and lower baseline/background IDUA activity, and therefore should not be interpreted as establishing mechanism from the present dataset alone. These data should be interpreted as pharmacodynamic proof-of-concept rather than definitive evidence of durable efficacy, as the genotype-stratified cohorts were small and no behavioral or organ-level disease endpoints were assessed.

Retrospective analysis of clinical trial samples revealed on-target effects: increased CEBPA protein correlated with higher IDUA levels in monocytes, and >50% of patients showed elevated plasma IDUA activity. Our findings suggest that RNA activation (RNAa) using MTL-CEBPA may help address this therapeutic gap by enabling reversible and titratable augmentation of endogenous IDUA production from engrafted donor cells without altering the genome. We therefore view MTL-CEBPA not as an alternative to effective hematopoietic gene therapy, but as a complementary strategy for patients in whom further adjustment of IDUA activity may be beneficial after transplantation. In contrast to *ex vivo* lentiviral IDUA gene transfer, RNAa does not require additional stem cell harvesting or reinfusion and does not result in permanent vector integration. This distinction may be particularly relevant for HSCT-treated Hurler patients, in whom a non-integrating, pharmacologically adjustable approach could usefully complement existing hematopoietic therapies. Further, RNAa enables IDUA expression to be modulated over time, with therapy tailored to the patient’s evolving clinical needs –allowing it to be increased, reduced, paused or discontinued– rather than solely committing the patient to a fixed level of transgene expression set at the time of transplantation.

Therapeutic oligonucleotides have already established a paradigm for addressing urgent, high-burden clinical needs in rare disease and systemic amyloidosis. Agents such as nusinersen and patisiran demonstrated that RNA-targeting drugs can be deployed at meaningful scale, with repeat dosing, within complex healthcare systems. The COVID-19 pandemic further underscored how rapidly nucleic acid–based therapies can be manufactured and globally distributed when an unmet need is clear. MTL-CEBPA and related RNAa therapeutics build on this foundation, but with a distinct mechanism upregulating rather than silencing target genes and here we show how this can be harnessed to tune enzyme production in MPS I-H.

Looking ahead, we envisage MTL-CEBPA being used as a long-term adjuvant to HSCT in Hurler syndrome and related MPS I-H subtypes, with intermittent IV dosing to maintain IDUA activity above a predefined functional threshold. In practice, this would allow clinicians to titrate enzyme elevation using a familiar pharmacology paradigm adjusting dose and frequency according to biomarkers and clinical status rather than relying on a one-time genetic intervention. The reversibility of RNAa-mediated transcriptional activation is a critical safety feature: if adverse effects emerge, dosing can be reduced or stopped, and expression is expected to return toward baseline over time. Such a strategy aligns with the lifelong nature of these disorders and offers a clinically intuitive framework for precision dosing in enzyme deficiency disease.

## Conclusion

The collective evidence presented here supports MTL-CEBPA as a promising adjuvant strategy for HSCT-treated MPS I-H, especially in patients with residual disease burden following transplantation. Its unique value lies in enabling reversible, adjustable enhancement of endogenous IDUA production from engrafted donor cells without permanent genome modification. This defines a clear therapeutic niche for RNA activation: not as a replacement for established HSCT or lentiviral gene therapy, but as a complementary, non-integrating approach that may allow enzyme activity to be pharmacologically fine-tuned over time according to clinical need. Together with the manufacturing scalability and cost profile of therapeutic oligonucleotides, these features position RNAa as a clinically realistic precision-dosing modality for Hurler syndrome and potentially other single-gene enzyme deficiency disorders. Future clinical studies should focus on defining dosing strategies that optimize durability, functional benefit, and safety in HSCT-treated patients

### Limitations

This work has several important limitations. First, the preclinical studies were designed primarily to establish pharmacodynamic proof-of-concept and therefore did not incorporate systematic evaluation of GAG reduction, detailed tissue pathology (e.g., bone, cartilage, heart valves, cornea) or functional endpoints in the MPS I-H models. Second, genotype-stratified cohorts in the humanized mouse experiments were relatively small, and the clinical analyses relied on retrospective, incompletely sampled biospecimens from oncology trials, limiting our ability to define precise exposure–response relationships. As a result, we cannot directly extrapolate the approximately two-fold increases in IDUA activity observed here to specific clinical benefits in Hurler syndrome. Future, prospectively designed studies in HSCT-treated MPS I-H patients, with predefined biochemical, imaging and functional readouts, will be required to determine the degree of enzyme augmentation needed for clinically meaningful improvement and to refine dose-finding strategies for MTL-CEBPA.

## Data Availability

The original contributions presented in this study are included in the article/Supplementary material, further inquiries can be directed to the corresponding authors.

## Footnotes

1
https://gene-regulation.com/pub/programs.html#match

2
https://eu.idtdna.com/site/order/designtool/index/GENOTYPING_PREDESIGN

3
www.idtdna.com/rhAmp-SNP-protocol
