# Supplementary material for: RNA activation as a precision dosing modality: MTL-CEBPA for controlled enzyme elevation in MPS I-H
Source: Front Med (Lausanne). 2026 May 7;13:1813362. doi: 10.3389/fmed.2026.1813362 (PMC13189782; doi:10.3389/fmed.2026.1813362)
Supplement: Supplementary file 1 [file Presentation_1.pptx]

## Slide 1
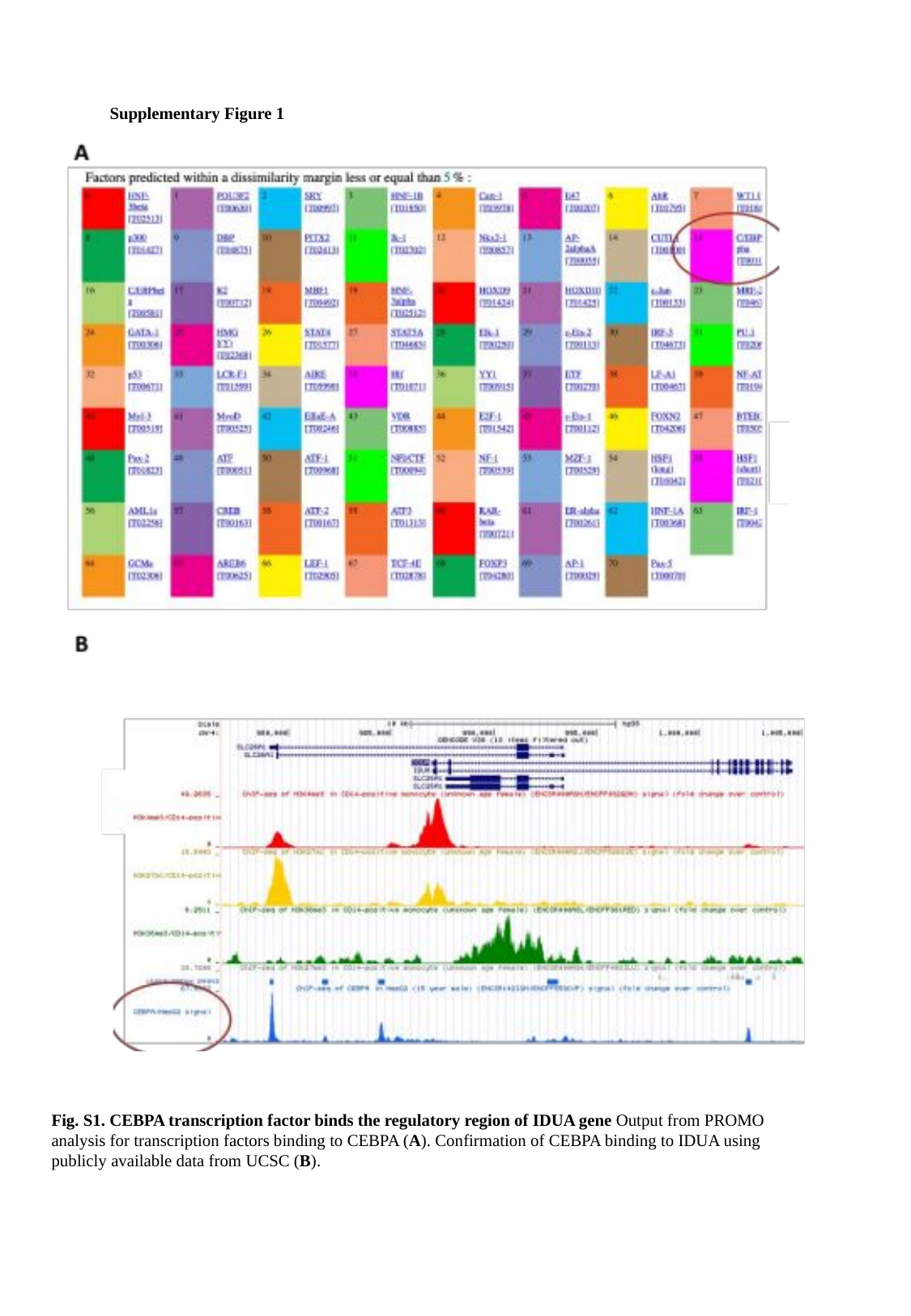

Supplementary Figure 1
Fig. S1. CEBPA transcription factor binds the regulatory region of IDUA gene Output from PROMO analysis for transcription factors binding to CEBPA (A). Confirmation of CEBPA binding to IDUA using publicly available data from UCSC (B).

## Slide 2
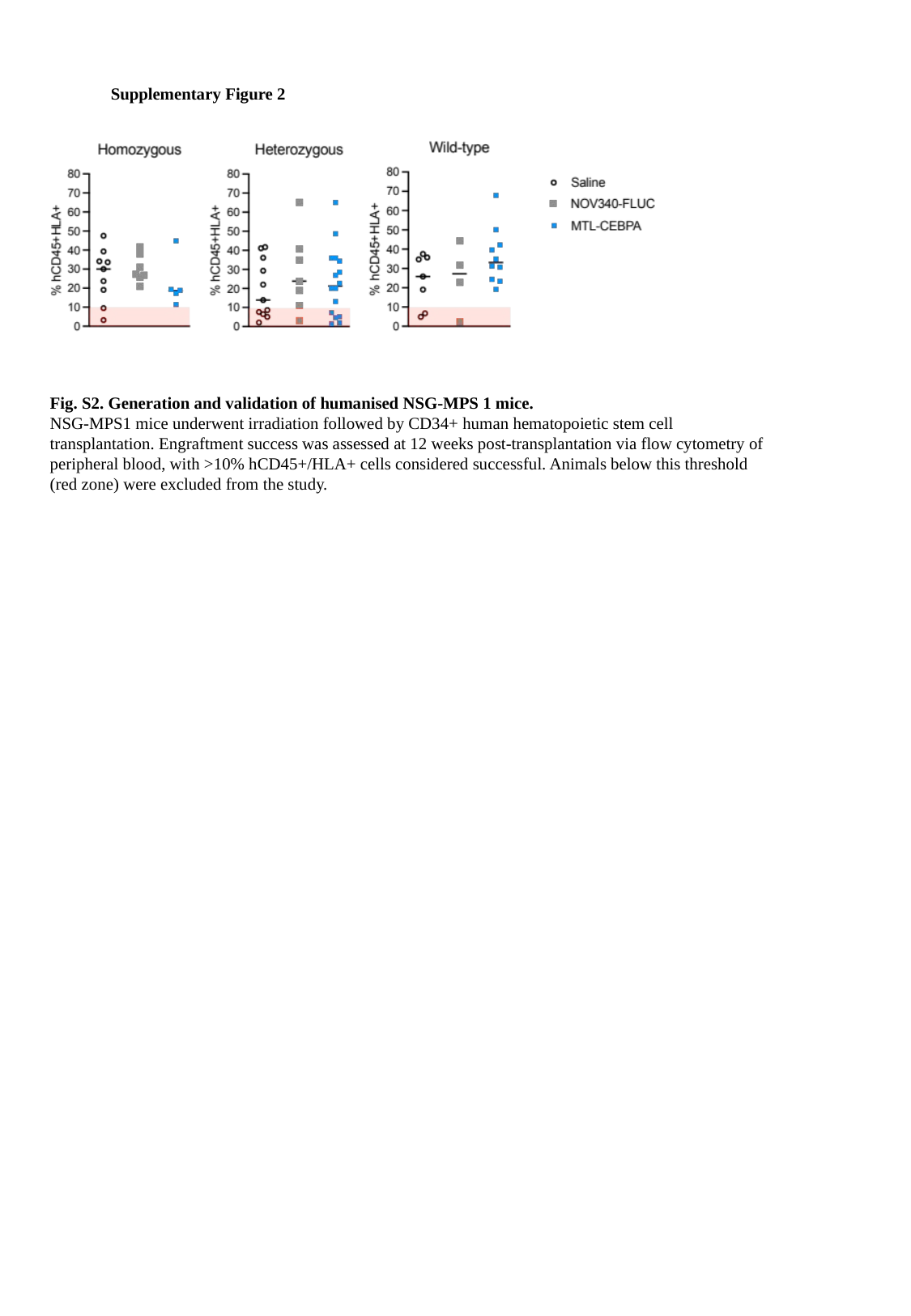

Supplementary Figure 2
Fig. S2. Generation and validation of humanised NSG-MPS 1 mice.
NSG-MPS1 mice underwent irradiation followed by CD34+ human hematopoietic stem cell transplantation. Engraftment success was assessed at 12 weeks post-transplantation via flow cytometry of peripheral blood, with >10% hCD45+/HLA+ cells considered successful. Animals below this threshold (red zone) were excluded from the study.
